# AREAdata: A worldwide climate dataset averaged across spatial units at different scales through time

**DOI:** 10.1016/j.dib.2022.108438

**Published:** 2022-07-02

**Authors:** Thomas P. Smith, Michael Stemkovski, Austin Koontz, William D. Pearse

**Affiliations:** a The Georgina Mace Centre for the Living Planet, Department of Life Sciences, Silwood Park Campus, Imperial College London, Buckhurst Road, Ascot, SL5 7PY, UK; b Department of Biology & Ecology Center, Utah State University, 5305 Old Main Hill, Logan, UT 84322, USA; c Center for Tree Science, Morton Arboretum, 4100 IL-53, Lisle, IL 60532, USA

**Keywords:** Climate, Temperature, Humidity, UV-radiation, Precipitation

## Abstract

In an era of increasingly cross-discipline collaborative science, it is imperative to produce data resources which can be quickly and easily utilised by non-specialists. In particular, climate data often require heavy processing before they can be used for analyses. Here we describe AREAdata, a continually updated, free-to-use online global climate dataset, pre-processed to provide the averages of various climate variables across different administrative units (*e.g.*, countries, states). These are daily estimates, based on the Copernicus Climate Data Store’s ERA-5 data, regularly updated to the near-present and provided as direct downloads from our website (https://pearselab.github.io/areadata/). The daily climate estimates from AREAdata are consistent with other openly available data, but at much finer-grained spatial and temporal scales than available elsewhere. AREAdata complements the existing suite of climate resources by providing these data in a form more readily usable by researchers unfamiliar with GIS data-processing methods, and we anticipate these resources being of particular use to environmental and epidemiological researchers.

**Specifications Table**

**Table:** 

Subject	Earth and Planetary Sciences
Specific subject area	Spatially averaged daily climate estimates
Type of data	Tables
How data were acquired	Downloaded from online repositories, then processed via a GIS methods pipeline.
Data format	analysed
Description of data collection	Raw, gridded climate rasters (temperature, specific humidity, relative humidity, UV-radiation and precipitation) are acquired from the Copernicus Climate Data Store. A raw population density raster was acquired from the Gridded Population of the World collection, version 4, revision 11. Downscaled CMIP6 future climate projections were acquired from WorldClim. We then process these raw data through our GIS methods pipeline to produce flat files with daily climate estimates for different spatial units, based upon shapefiles acquired from the Global Administrative Areas (GADM) database. Periodically, new climate data are automatically downloaded and processed and the output files updated.
Data source location	Primary data sources: Copernicus Climate Data Store: https://cds.climate.copernicus.eu/ Gridded Population of the World: https://sedac.ciesin.columbia.edu/data/set/gpw-v4-population-density-rev11 WorldClim: https://www.worldclim.org/ GADM: https://gadm.org/
Data accessibility	Repository name: figshare Data identification number: 16587311 & 16770004 Direct URL to data: https://figshare.com/articles/dataset/AREAdata_GID2_output_files/16587311https://figshare.com/articles/dataset/AREAdata_static_output_files/16770004
Related research article	T.P. Smith, S. Flaxman, A.S. Gallinat, S.P. Kinosian, M. Stemkovski, H.J.T. Unwin, O.J. Watson, C. Whittaker, L. Cattarino, I. Dorigatti, M. Tristem, W.D. Pearse, Temperature and population density influence SARS-CoV-2 transmission in the absence of nonpharmaceutical interventions. Proc. Natl. Acad. Sci. USA. 118:25 (2021) e2019284118. https://doi.org/10.1073/pnas.2019284118

## Value of the Data

• AREAdata provides estimates of daily climate data, population density, and future climate forecasts, averaged across different spatial units at different scales, distributed in easy to use file formats.
• We believe these data are of wide use, but specifically we see use-cases for ecologists and epidemiologists. In particular, researchers untrained in GIS methods would benefit from the accessible nature of how we distribute these data.
• We have already used these data to investigate the seasonality of SARS-CoV-2 (the causative agent of COVID-19) [1], [2] and envisage further use of these data for understanding the seasonal responses of infectious diseases. Furthermore, the continually updating nature of this dataset makes it particularly useful for for rapid analyses in response to new disease emergence.
• Many other researchers have applied similar methods to the same underlying data in order to quantify climate variables, resulting in a mass duplication of effort [3], [4], [5], [6], [7], [8]. By using AREAdata, this duplication of effort could be reduced.
• Climate datasets are essential for researchers across many disciplines, however are generally available only in formats that require extensive processing and specialist knowledge to use. AREAdata makes climate data accessible and open to non-specialists.

## 1 Data Description

AREAdata can be accessed at our *GitHub* site (https://pearselab.github.io/areadata), which contains download links to each data file. The data are also released on figshare (daily climate updates: https://doi.org/10.6084/m9.figshare.16587311; static population density and future annual mean temperatures: https://doi.org/10.6084/m9.figshare.16770004).

These are distributed both as .RDS files for use in the R statistical programming environment and as zipped tab-delimited files for other uses. Details of each file are given in Table 1. The daily climate files consist of a matrix of point estimates of an environmental variable (either temperature, specific humidity, relative humidity, UV or precipitation), with rows representing each spatial unit that the variable was averaged across and columns representing the date. These daily files are periodically updated, by automatically downloading and processing new data as it becomes available. The population density files consist of a matrix with a single column of population density point estimates, with rows for each spatial unit. The climate forecast files consist of a matrix of point estimates for annual mean temperatures, with rows representing each spatial unit, and columns representing the combination of global climate model (GCM) and shared socio-economic pathway (SSP), and the year range of the projection. Column headers for the forecasting files follow the labelling convention <GCM>_<SSP>_<XXXX-YYYY>, where XXXX-YYYY specifies the date range of the forecast. These files are all distributed by the level of spatial organisation that the data have been averaged across (*i.e.* separate files for countries, states, counties). In the initial release, AREAdata provided daily climate estimates from 2020-01-01 to 2021-09-30.

**Table 1:** List of all files distributed by AREAdata. All files are available both in.RDS and zipped.txt formats (with filenames appended as such). Status column shows which files are released only once with this dataset (static), or are continuously updated when new data become available (updating). For the updating files, new data are periodically downloaded and processed, and the new estimates are appended to the old files and re-published with the same file-names. Publication of these data on figshare enables previous versions to also remain online and be downloaded alongside updated versions.

File name	Variable	Units	Areas	Status
temp-dailymean-countries-cleaned	temperature	${}^{\circ }$C	GID0 (countries)	updating
temp-dailymean-GID1-cleaned	temperature	${}^{\circ }$C	GID1 (states)	updating
temp-dailymean-GID2-cleaned	temperature	${}^{\circ }$C	GID2 (counties)	updating
spechumid-dailymean-countries-cleaned	specific humidity	kg kg${}^{-1}$	GID0 (countries)	updating
spechumid-dailymean-GID1-cleaned	specific humidity	kg kg${}^{-1}$	GID1 (states)	updating
spechumid-dailymean-GID2-cleaned	specific humidity	kg kg${}^{-1}$	GID2 (counties)	updating
relhumid-dailymean-countries-cleaned	relative humidity	%	GID0 (countries)	updating
relhumid-dailymean-GID1-cleaned	relative humidity	%	GID1 (states)	updating
relhumid-dailymean-GID2-cleaned	relative humidity	%	GID2 (counties)	updating
uv-dailymean-countries-cleaned	UV radiation	J m${}^{-2}$	GID0 (countries)	updating
uv-dailymean-GID1-cleaned	UV radiation	J m${}^{-2}$	GID1 (states)	updating
uv-dailymean-GID2-cleaned	UV radiation	J m${}^{-2}$	GID2 (counties)	updating
precip-dailymean-countries-cleaned	precipitation	m	GID0 (countries)	updating
precip-dailymean-GID1-cleaned	precipitation	m	GID1 (states)	updating
precip-dailymean-GID2-cleaned	precipitation	m	GID2 (counties)	updating
population-density-countries	population density	people km${}^{-1}$	GID0 (countries)	static
population-density-GID1	population density	people km${}^{-1}$	GID1 (states)	static
population-density-GID2	population density	people km${}^{-1}$	GID2 (counties)	static
annual-mean-temperature-forecast-countries	future temperature	${}^{\circ }$C	GID0 (countries)	static
annual-mean-temperature-forecast-GID1	future temperature	${}^{\circ }$C	GID1 (states)	static
annual-mean-temperature-forecast-GID2	future temperature	${}^{\circ }$C	GID2 (counties)	static

To ensure that those who process and release the raw data going into AREAdata are properly acknowledged, a condition of use of AREAdata is the citation of the raw data, and this information is provided on the website.

## 2 Experimental Design, Materials and Methods

To produce the daily climate estimates provided in AREAdata, we gather gridded rasters describing daily climate data and average these climate variables across the geographic areas of spatial units at different levels of administrative organisation.

Below, all software packages given in *italics* are *R* packages (version 4.1.0) [9] unless otherwise specified. The code to fully reproduce this pipeline is freely available under a GPL v3.0 license and can be acquired from our *GitHub* repository (https://github.com/pearselab/areadata). An archived version of the code used in this publication is available on zeonodo (https://doi.org/10.5281/zenodo.5901419).

Continual updates of the output files as new climate data becomes available can be found on our *GitHub* project website (https://pearselab.github.io/areadata/) and on figshare (https://doi.org/10.6084/m9.figshare.16587311). These continual updates are automatically released monthly, however the underlying code to run these updates locally is also shared so that users can update these data to-the-day when necessary. Output files for the county-level estimates are large (>100MB), and so are released only on figshare. Data on either platform are version-controlled with dates of submission recorded and past versions archived.

Users can also create custom downloads for the county-level (GID2) data using an R Shiny app (https://smithtp.shinyapps.io/areadata-app/). This allows for finer control in which parts of the data are downloaded, rather than downloading these large files in their entirety.

Static output files for population density and future estimates of annual mean temperatures can also be found on our *GitHub* website and figshare (https://doi.org/10.6084/m9.figshare.16770004).

### 2.1 Data collection

We acquire shapefiles for worldwide administrative areas from the Global Administrative Areas (GADM) database [10] at three different spatial scales: GID 0, GID 1, and GID 2. GID 0 is equivalent to countries, and (in the USA) GID 1 and GID 2 are equivalent to states and counties respectively.

We collect hourly estimates of climatic variables for the ERA-5 reanalysis from the Coperincus Climate Change Service’s Climate Data Store (CDS). Temperature (K), specific humidity (kg kg${}^{-1}$; mass of water vapour per kilogram of moist air), and relative humidity (%; water vapour pressure as a percentage of the air saturation value) are acquired from the pressure-levels dataset [11] at 1000 hPa (*i.e.*, surface atmospheric pressure). Estimates of ultraviolet (UV) levels (J m${}^{-2}$; the amount of UV radiation reaching the surface) and precipitation (m; total precipitation, the accumulated liquid and frozen water falling to the Earth’s surface as measured in metres of water equivalent) are acquired from the surface-level dataset [12].

Global population density data are acquired from the Gridded Population of the World collection, version 4, revision 11 [13]. These data consist of population density estimates based on national and sub-national censuses and population registers. They use a gridding algorithm to assign population densities to grid cells, and these data are provided as rasters at different scales. Here we use the 15 arc-minute resolution for consistency with the resolution of the ERA5 climate data.

Downscaled CMIP6 future climate projections are acquired from WorldClim [14]. CIMP6 is the 6th phase of a global climate model (GCM) inter-comparison project, coordinating the design and distribution of global climate model simulations [15]. These model simulations are typically numerically complex and thus to facilitate fast computation, the world is divided into coarse grid cells. This is not ideal for studies investigating phenomena at higher spatial scales, and thus WorldClim provides downscaled versions of future predictions from GCM outputs, at higher spatial resolutions, based on WorldClim v2.1 as baseline climate. WorldClim provides these downscaled data for nine GCMs: BCC-CSM2-MR, CNRM-CM6-1, CNRM-ESM2-1, CanESM5, GFDL-ESM4, IPSL-CM6A-LR, MIROC-ES2L, MIROC6, MRI-ESM2-0, and for four Shared Socio-economic Pathways (SSPs): 126, 245, 370 and 585.

### 2.2 Climate averaging pipeline

We use the Climate Data Operators program [16] to compute daily means from the hourly data for each of the climate variables acquired from the CDS. We then calculate the mean value of each environmental variable across the administrative units given in each of our acquired shapefiles (*i.e.* countries, states, etc.), using the *exactextractr* R package. Specifically, we compute the mean of all grid cells fully or partially covered by the administrative unit polygon, weighted by the fraction of each cell covered by the polygon. When new climate data becomes available, these are appended to the previously extracted data to produce a single, live, updated output file for each administrative level and environmental variable combination. The data produced are simple files containing the daily climate estimates by spatial unit, *e.g.* country and by date, which we output as .RDS files for use in R and as zipped tab-delimited text files for other applications. We use an automated pipeline to produce new estimates on a monthly basis, which updates these files and automatically publishes new versions to *GitHub* and figshare (the links for which remain constant).

We use the same methods to process the gridded population density data, which we provide similarly with a single population density estimate for each spatial unit. We process annual mean temperatures from the climate forecast data, and again provide estimates by spatial unit for each combination of GCM and SSP. The population density and temperature forecast output files are static (not continually updated). Our website provides an easy interface to download these data; however, users can also run the provided code locally to make adjustments to the calculations and generate their own files.

## Ethics Statement

Not applicable - no human or animal subjects used in the generation of this dataset.

## CRediT authorship contribution statement

**Thomas P. Smith:** Conceptualization, Methodology, Software, Validation, Writing – original draft, Writing – review & editing. **Michael Stemkovski:** Methodology, Software, Writing – review & editing. **Austin Koontz:** Software, Writing – review & editing. **William D. Pearse:** Conceptualization, Methodology, Supervision, Writing – review & editing.

## Declaration of Competing Interest

The authors declare that they have no known competing financial interests or personal relationships which have, or could be perceived to have, influenced the work reported in this article.

## Data Availability

AREAdata updating output files (Original data) (Mendeley Data).
